# Pleomorphic Adenoma with Epithelial Atypia, Apocrine Metaplasia, and/or In situ/Intracapsular Salivary Duct Carcinoma Are Indolent Lesions with Good Prognosis: A Proposal for Unified Nomenclature and Clinical Observation

**DOI:** 10.1007/s12105-025-01841-8

**Published:** 2025-09-12

**Authors:** Grayson G. Cole, Matt Levin, David Ferber, Spencer C. Roark, Peter M. Sadow, Daniel Lubin, Julie Guilmette, Jason R. Pettus, Adam S. Fisch, Dipti P. Sajed, Fouad R. Zakka, Mark W. Lingen, Nicole A. Cipriani

**Affiliations:** 1 Department of Pathology, University Hospitals, Cleveland, OH USA; 2 Cell IDx, Inc. (A Division of Leica Biosystems), San Diego, CA USA; 3 Medical & Scientific Affairs, Leica Biosystems, Deer Park, IL USA; 4 Vanderbilt University Medical Center, Nashville, TN USA; 5 Department of Pathology, Massachusetts General Hospital, Harvard Medical School, Boston, MA USA; 6 Emory Healthcare, Atlanta, GA USA; 7 Hôpital Charles-Le Moyne, Québec, QC USA; 8 Dartmouth-Hitchcock Medical Center, Lebanon, NH USA; 9 Department of Pathology, University of California, Los Angeles, Los Angeles, CA USA; 10 Wellstar Health System, Atlanta, GA USA; 11 Department of Pathology, The University of Chicago, Chicago, IL USA

**Keywords:** In situ, Intracapsular, Salivary duct carcinoma, Dysplasia, Atypical epithelial cells, AR, HER2, p40

## Abstract

**Purpose:**

Salivary duct carcinoma (SDC) is the most common malignancy to arise in a pleomorphic adenoma (PA). Intracapsular or non-invasive carcinoma ex PA (CXPA) is defined by the presence of malignant-appearing tumor cells within the PA that do not violate the tumor border. Knowledge regarding the possible morphologic spectrum and prognosis of intratumoral CXPA is scarce. This study aims to evaluate the morphologic, immunohistochemical, and clinical features of PAs with apocrine / salivary-duct-like intratumoral atypia.

**Methods:**

Surgical pathology databases were queried for in situ or intracapsular/intratumoral SDC ex PAs and PAs with atypical epithelial cells (AEC). Exclusion criteria included recurrent lesions, invasion, positive margins, atypia only in myoepithelial cells, or other morphologic variants. Chromogenic multiplex (androgen receptor (AR) and HER2) and monoplex (p40) assays were performed on all available cases, as well as on a control group of non-atypical benign PAs and overtly invasive SDCs. Clinical outcomes were recorded.

**Results:**

96 cases were identified: 23 AEC, 6 apocrine metaplasia, 41 benign PA, 8 SDC ex PA. All AEC, apocrine metaplasia, and benign cases were treated with surgery alone, with 3 AEC cases also receiving a neck dissection. No case recurred. Five of 8 SDC ex PA recurred; 3 died of disease. AR and HER2 were respectively expressed in 96% and 22–48% of AEC; 83% and 0% of apocrine metaplasia; 51% and 0% of benign PA; and 86–100% and 38–57% of SDC ex PA. Patients had increasing average age from benign (~ 50 years) to atypical/in situ (60 years) to invasive carcinoma (~ 70 years).

**Conclusion:**

The presence of epithelial atypia within a PA (ranging from isolated AR expression to apocrine metaplasia to overtly dysplastic/malignant epithelial cells) does not portend recurrence or metastasis if the atypia is confined within the borders of the adenoma and negative margins are achieved. Therefore, use of the term “in situ/intracapsular/intratumoral salivary duct carcinoma ex pleomorphic adenoma” is discouraged in light of good prognosis and potential for overtreatment by clinical teams. Nomenclature such as pleomorphic adenoma with epithelial “atypia” or “dysplasia” is recommended, followed by a comment regarding the morphologic features and likely indolent behavior.

## Purpose

Carcinoma ex pleomorphic adenoma (CXPA) was first recognized as a distinct entity in the 2005 WHO classification [1]. It is now recognized that CXPA is composed of a variety of different carcinomas that may arise in pleomorphic adenomas (PAs). Subclassification of the CXPA is a necessary part of diagnosis as salivary gland malignancies have a spectrum of prognoses [2–4]. Salivary duct carcinoma is the most common malignancy to arise in CXPA and is usually a high-grade carcinoma with poor prognosis [2, 5].

CXPA poses a histologic challenge as even benign PAs can show concerning features including pseudopod-like penetration beyond the capsule/border of the lesion, lobular growth, infarct-type necrosis, degenerative nuclear atypia, hypercellularity, and biopsy-related changes. PAs may not show a distinct fibrous capsule, as often seen in the palate. Metaplastic change can occur within the epithelial or stromal compartments, including squamous, sebaceous, mucinous, adipocytic, osseous, chondroid, and (less often) ceruminous or trichilemmal [6–8]. Moreover, the myoepithelial cell population is notoriously varied, and can be seen as spindled, stellate, plasmacytoid, epithelioid, clear, oncocytic, and even schwannoma-like [6, 9]. Apocrine metaplasia of epithelial cells is a phenomenon that has been identified in pleomorphic adenomas with relatively limited discussion when compared to other recognized metaplasias [10–12].

What constitutes a malignant diagnosis has been subject to debate since the definition of CXPA. Stratification of malignant progression from within to outside the PA is recognized in the form of in situ or intraductal intracapsular, extraductal intracapsular, minimally invasive, and widely invasive CXPA. Intracapsular or non-invasive CXPA is defined by the presence of malignant tumor cells within the PA that do not violate the tumor border. As a capsule may not be uniformly present, “intracapsular” will interchangeably be referred to as “intratumoral” [6, 13, 14]. This category has further been stratified into in situ or intraductal intratumoral (in which a myoepithelial layer is retained) versus extraductal intratumoral (in which a myoepithelial layer is not retained). Authors have also suggested a category of intraductal extracapsular/extratumoral CXPA, in which the invasive CXPA outside the borders of the tumor has a retained myoepithelial cell layer [15].

Initially a threshold of 1.5 mm of extratumoral invasion was utilized to define minimally invasive, however data has shown that up to 4–6 mm seem to have a similar prognosis [3]. These lesions typically behave well but metastases and recurrence are reported [3, 15, 16]. Widely invasive CXPA is defined as any tumor that has greater than or equal to 6 mm extratumoral invasion and has worse outcomes [17].

While there is good evidence to support minimally and widely invasive CXPA as entities with distinct prognoses and treatment indications, knowledge regarding intratumoral CXPA is relatively lacking. The current literature characterization of intratumoral CXPAs has found they can show necrosis and increased mitotic activity [18]. Expression of androgen receptor (AR), HER2, and GCDFP-15 have been reported [18–20]. BAP1 may be lost [21]. While the landscape of molecular alterations within CXPAs is as complex as the spectrum of malignancies it contains, CXPAs often retain the PLAG/HMGA2 alterations from the originating pleomorphic adenoma [22–24]. CXPAs may also show characteristic molecular alterations associated with the subtype of salivary gland carcinoma similar to the de novo counterpart [22, 25].

While this characterization has allowed a somewhat clearer picture of the current definitions, it does not address the questions: What is the morphologic spectrum of and histologic cutoffs for “atypia,” “dysplasia,” or “carcinoma-in situ” within a pleomorphic adenoma? Do any of these morphologic changes predict malignant behavior? What is the threshold for diagnosis of intratumoral/in situ carcinoma? The prognostic implications and subsequent treatment recommendations for intratumoral atypia/carcinoma with negative surgical margins has been a contentious subject. Evidence has shown that benign PAs have a low recurrence rate, particularly if surgical margins are negative [26]. As intratumoral CXPA are relatively uncommon, outcome data is limited. However, studies have shown that intratumoral CXPA may share this indolent prognosis if margins are negative [3, 15, 19, 20].

As salivary duct carcinoma (SDC) ex PA is one of the most common malignancies to arise from the epithelial compartment of pleomorphic adenomas, this study aims to evaluate the morphologic, immunohistochemical, and clinical features of pleomorphic adenomas with apocrine / salivary-duct-like atypia. In order to fully grasp the prognostic implications of these lesions, this study also investigates clinical outcomes and conducts a review of the current literature with a focus on follow up data.

## Methods

### Clinical Case Collection and Review

Surgical pathology databases from the authors’ institutions were queried for cases of in situ or intracapsular/intratumoral salivary duct carcinoma-ex PAs and PAs with atypical epithelial cells (AEC) from 1/1/2000-9/1/2023. Institutions included: The University of Chicago; Massachusetts General Hospital; Emory Healthcare; Hôpital Charles-Le Moyne, Québec; Dartmouth-Hitchcock Medical Center; University of California, Los Angeles; and Northside Hospital. Institutional Review Board Approval was obtained at each institution.

As there is no clear consensus on the different morphologic definitions of atypia, dysplasia, or in situ CXPA, a wide net was cast and these entities are included in a single cohort of PAs with AEC: epithelial cells that showed features such as significant nuclear or cellular enlargement, hyperchromatic nuclei, irregular nuclear contours, coarse chromatin, prominent nucleoli, anisonucleosis, mitoses, or association with tumor necrosis. Foci of cells with abundant granular eosinophilic cytoplasm, luminal snouting, and moderately enlarged ovoid nuclei with nucleoli (but without nuclear hyperchromasia, anisonucleosis, > 1 mitosis/2 mm^2^, or necrosis) were defined as apocrine metaplasia. Exclusion criteria included recurrent lesions, invasion, positive margins, atypia present only in myoepithelial cells, or other morphologic variants.

All cases were subject to histologic review (NAC, GC, SR) if materials were available. Authors evaluated whether the atypia met above criteria for AEC. Cases with squamous metaplasia and increased myoepithelial cellularity without atypical/malignant features as described above were included as benign PAs. Cases that met criteria for apocrine metaplasia were separated.

A positive control cohort of widely invasive SDC ex PA was identified in the University of Chicago archives (1990–2023). Similarly, a negative control cohort of benign, non-atypical PAs was also identified (1990–2000). The negative control cohort had exclusion criteria of recurrent lesions and positive margins.

#### Immunohistochemical Staining

All tissues were processed for routine histopathologic diagnosis following resection, specifics depending on institution of origin. A select representative block per case was chosen. All cases were formalin-fixed paraffin-embedded (FFPE) whole tissue sections. Chromogenic multiplex (androgen receptor and HER2) and monoplex (p40) assays were performed at UChicago for all available cases.

Chromogenic multiplex assay (androgen receptor, HER2): FFPE sections were stained with anti-human primary antibodies, conjugated to unique epitope UltraTags (performed at Cell IDx, San Diego). Slides were baked in a 60 °C oven for one hour, and for each tissue section tested, heat-induced epitope retrieval (HIER) was performed on the Leica Biosystems BOND RX Research Stainer using ER2 (pH 9.0) for 20 min. After a 5-minute peroxide block, a 3% rabbit serum protein block was applied for 20 min. Directly afterward, the primary anti-human tagged panel consisting of anti-human androgen receptor (Clone EPR1535(2), Abcam, Cambridge, MA) and HER2 (Clone EP1045Y Abcam, Cambridge, MA), were assembled into a cocktail at pre-determined concentrations and incubated on tissue sections for 30 min. After the proscribed washing steps, the secondary enzyme-conjugated anti-Tag antibody cocktail was applied to slides for 30 min, followed by assigned chromogen deposition.

Chromogenic monoplex assay (p40): Serial FFPE sections had identical pre-treatment as the multiplex slides before application of the primary antibody. Anti-human p40 (Clone EPR17863-47, Abcam, Cambridge, MA) was stained using Protocol F on the Leica Biosystems BOND RX Research Stainer followed by the Bond Polymer Define Detection Kit.

AR was interpreted on a binary scale, with any expression in AEC regarded as positive. HER2 was interpreted according to ASCO/CAP recommendations for scoring of breast carcinoma on a scale of 0, 1+, 2+, and 3 + in AEC [27]. HER2 staining of 0 or 1 + was regarded as negative and staining of 2 + or 3 + was regarded as positive. Presence or absence of p40 was documented as a marker of myoepithelial cell presence or absence surrounding AECs. In the positive control group of SDC ex PA, the intracapsular/in situ epithelial component and the invasive component were evaluated separately using the same criteria. In the negative control group of cases of non-atypical PAs, the non-atypical ductal elements were evaluated using the same criteria.

### Literature Review

A literature review was conducted by searching PubMed and google scholar for the terms “pleomorphic adenoma with atypia,” “pleomorphic adenoma carcinoma in situ,” and “intracapsular carcinoma ex pleomorphic adenoma.” Only cases of pleomorphic adenomas of the head and neck with intracapsular carcinoma or epithelial atypia with follow up data were included.

## Results

### Clinical Case Review (Table 1)

**Table 1:** Clinicopathologic parameters

Parameter	PA with AEC (*n* = 23)	PA with apocrine metaplasia* (*n* = 6)	PA control (*n* = 41)	SDC control (*n* = 8)
Sex Ratio (M: F)	1.9	1	0.71	7
--Male	15	3	17	7
--Female	8	3	24	1
Age at presentation (average)	60	57.5	49	69
Location	–	–	–	–
--Parotid	16	6	29	6
--Submandibular gland	3	0	9	1
--Parapharyngeal space/deep parotid	3	0	2	1
--Minor salivary glands	1	0	1	0
Tumor size (average, cm)	3.0	3.2	2.4	2.5
Necrosis present	6	0	1*	4
Mitoses > 1 / 2 mm^2^	8	0	0	6
Treatment	–	–	–	–
--Surgery alone	23	6	41	1
--Surgery with adjuvant radiation	0	0	0	7
Follow up (average, years)	2.6	8.9	10.5	3.7
Recurrences	0	0	0	5

PA = pleomorphic adenoma, AEC = atypical epithelial cells, SDC = salivary duct carcinoma, *infarct-type necrosis

**Fig. 1:**
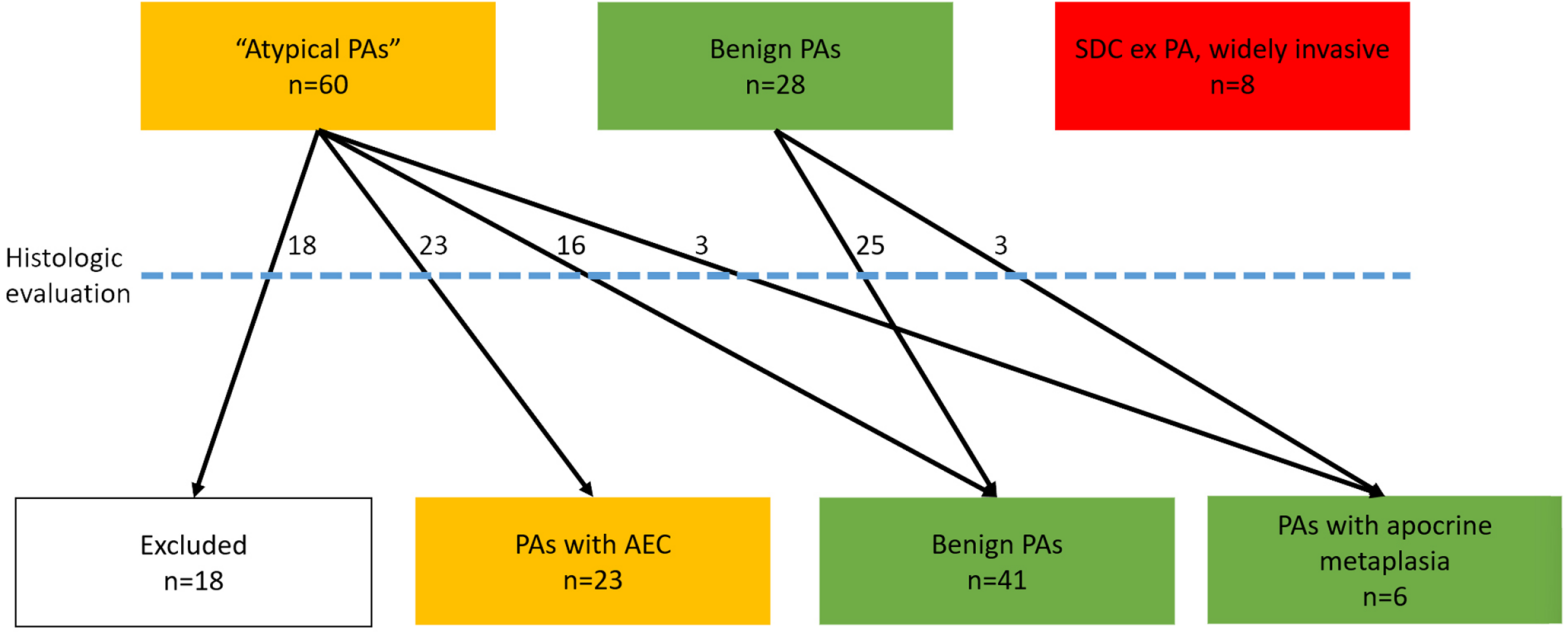
Flow chart showing identified cases, carried through consensus histologic evaluation, to final status. Key: PA = pleomorphic adenoma, SDC = salivary duct carcinoma, AEC = atypical epithelial cells

A total of 96 cases were identified: 23 met criteria for AECs, 6 for apocrine metaplasia, 41 for benign PA (including 7 with benign squamous metaplasia, 10 cellular/myoepithelial-rich, 1 with infarct-type necrosis, 1 stromal-rich), and 8 for SDC ex PA (Fig. 1). Additionally, 18 other cases were regarded as atypical by the contributing pathologist but did not meet inclusion criteria: now-recognized HMGA2-like morphology (*n* = 6), myoepithelial atypia (*n* = 4), myoepithelial carcinoma ex PA (*n* = 2), tumor not present/exhausted (*n* = 3), and other miscellaneous (*n* = 3).

**Fig. 2:**
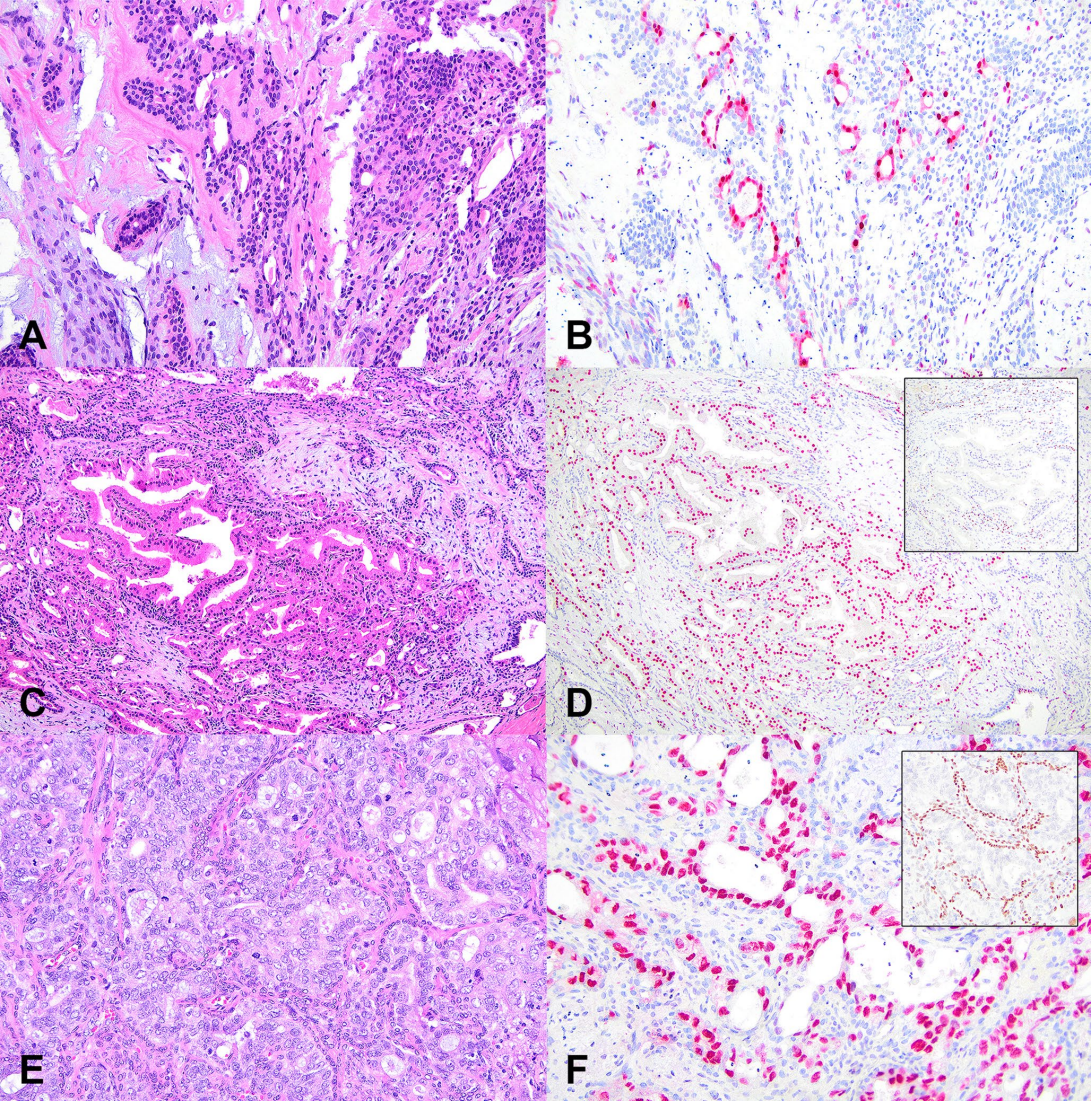
Histologic images of bland PA, apocrine metaplasia, and atypical PA. This conventional pleomorphic adenoma with no cytologic atypia **A** shows focal androgen receptor expression **B** in bland luminal epithelial cells. This case of apocrine metaplasia **C** shows brightly eosinophilic cells with luminal snouts. They express androgen receptor **D** and are surrounded by p40-positive basal/myoepithelial cells (D inset). This atypical pleomorphic adenoma shows a proliferation of atypical epithelial cells with large vesicular nuclei and numerous mitoses **E**. Atypical cells express androgen receptor **F** and are surrounded by p40-positive basal/myoepithelial cells (intratumoral / intraductal pattern) (F inset)

There were 23 cases meeting criteria for AEC (Table 1). Males were more frequently affected than females. The average age was 60 (23–80) years. Parotid was the most frequent anatomic site. Average size was 3.0 (1.3-6) cm. Six cases had necrosis (26%). Eight cases showed > 1 mitosis/2 mm^2^ (35%). The highest mitotic count (20/2 mm^2^) was in a tumor in which the atypical component occupied ~ 50% of the lesion (Fig. 2E). The largest extent of atypia was in a tumor completely occupied (100%) by atypia. In both cases, the atypia was intraductal (p40 + myoepithelial cells present). All tumors had negative margins. All cases were treated with surgery alone, with 3 cases additionally receiving a neck dissection. Follow up ranged from 0 to 12 (average 2.6, median 0.9) years. No case recurred.

There were 6 cases with apocrine metaplasia (Table 1). Males and females were equally affected. The average age was 57.5 (24–76) years. Parotid was the only involved site. Average size was 3.2 (1.1–5.1) cm. No tumor had necrosis or mitoses. All had negative margins. All cases were treated with surgery alone. Follow up ranged from 0 to 21 (average 8.9, median 7.6) years. No case recurred.

There were 41 cases of benign pleomorphic adenomas (Table 1). Females were more frequently affected than males. The average age was 48 (13–95) years. Parotid was the most frequent anatomic site. Average size was 2.4 (0.9–7.6) cm. No tumor had mitoses; none had true tumor necrosis (one had infarct-type necrosis). All had negative margins. All cases were treated with surgery alone. Follow up ranged from 0 to 21.8 (average 10.3, median 14) years. No case recurred.

There were 8 cases of widely invasive SDC ex PA (Table 1). Males were more frequently affected than females. The average age was 69 (52–87) years. Parotid was the most frequent anatomic site. Average size was 2.5 (1.3–4.8) cm. A positive margin was present in 1 case. One case did not have an appreciable in situ component on the available slide (however, a burnt-out pleomorphic adenoma was described in the report). Six cases had > 1 mitosis/2 mm^2^ and 4 had necrosis. One case was treated with surgery alone, and another was treated with surgery and adjuvant radiation. The remaining six patients received adjuvant chemotherapy and radiation. Five cases recurred, with an average time to recurrence of 1.3 years. Three died from disease, with an average time from initial diagnosis to death of 2.3 years. Average follow up of alive patients was 4.5 (median 3.5) years.

Overall, atypical pleomorphic adenomas and SDC ex PA occurred more frequently in males (2:1 and 7:1 M: F ratio, respectively). Atypical PAs occurred in patients on average 10 years older than benign PAs; SDC ex PAs occurred in patients on average 10 years older than atypical PAs. These data suggest two decades of progression from benign to atypical/in situ to invasive carcinoma, with an increased frequency in males.

Of the 18 excluded cases that were called atypical by the original pathologist (but did not meet criteria for AEC, apocrine metaplasia, benign, or overt SDC ex PA), all were surgically resected and 3 received adjuvant radiotherapy. Follow up ranged from 0 to 22.2 (average 5.5, median 3.9) years. No case recurred.

### Immunohistochemical Findings (Table 2)

**Table 2:** Immunohistochemical findings

Tumor Type	p40 retained (%)	AR expressed (%)	HER2 2 + or 3+ (%)
PA with AEC (*n* = 23)	21 (91)	22 (96)	5 (22)
PA with apocrine metaplasia (*n* = 6)	6 (100)	5 (83)	0 (0)
PA control (*n* = 41)	41 (100)	21 (51)	0 (0)
SDC control: in-situ component (*n* = 7)	4 (57)	6 (86)	4 (57)
SDC control: invasive component (*n* = 8)	1 (13)	8 (100)	3 (38)

PA = pleomorphic adenoma, AEC = atypical epithelial cells, SDC = salivary duct carcinoma

**Fig. 3:**
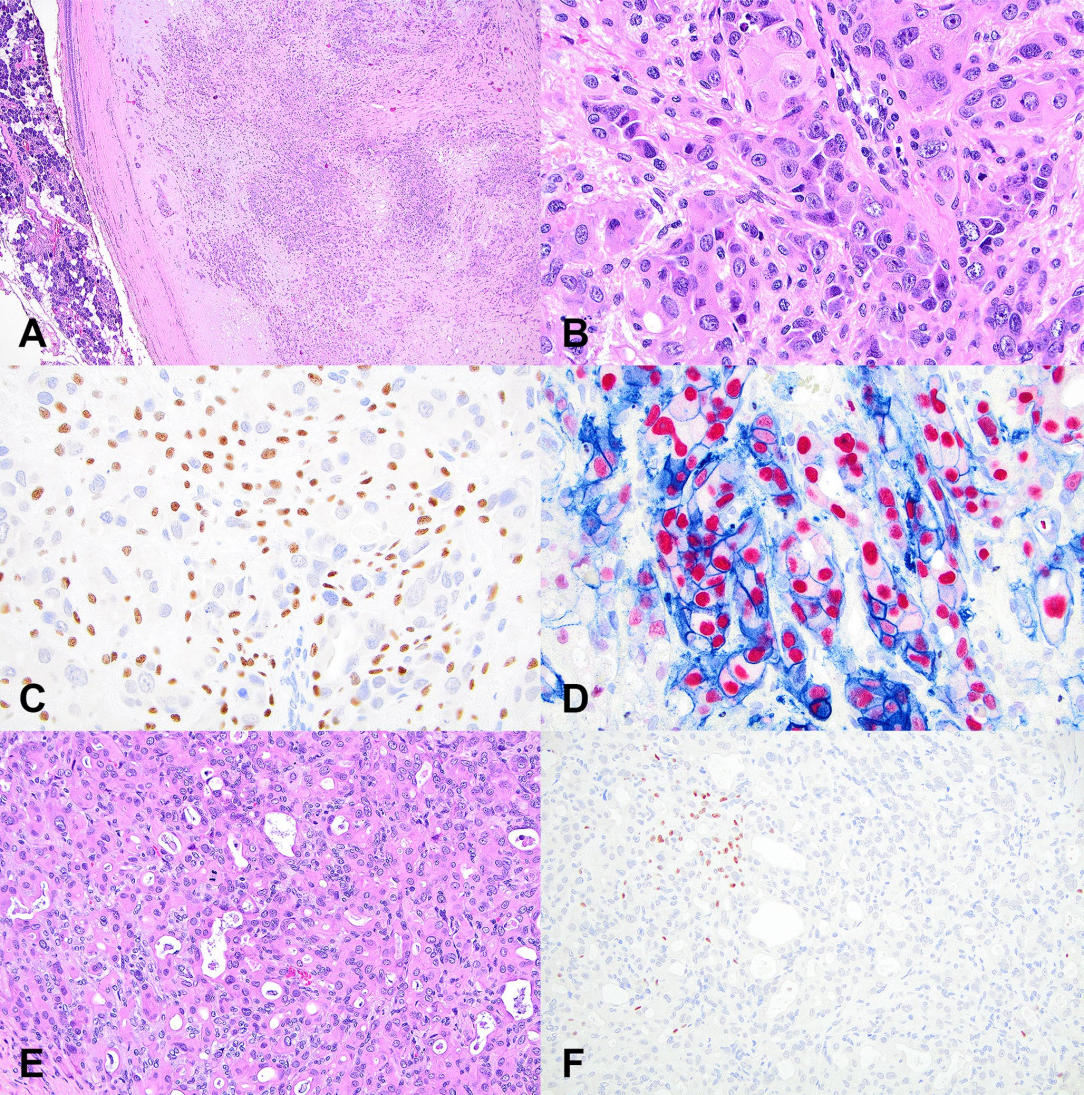
This pleomorphic adenoma with overt epithelial atypia is circumscribed on low power with no invasion **A**. Focally, luminal epithelial cells are enlarged with irregular nuclei and prominent nucleoli **B**. They are surrounded by a p40-positive basal/myoepithelial cell layer **C**. However, they strongly express nuclear androgen receptor (red) and membranous HER2 (blue)(**D**. In other areas, atypical epithelial cells are clustered, harbor mitoses **E**, and are not uniformly surrounded by basal/myoepithelial cells on p40 stain (intratumoral / extraductal pattern) **F**

Most PAs with AECs had retained p40-positive myoepithelial cells in areas of epithelial atypia (*n* = 21/23, 91%). Two cases (9%) showed focal loss of p40 in atypical areas (Fig. 3). However, these areas remained within the confines of the neoplasm. Androgen receptor was expressed in the AECs in most cases (*n* = 22/23, 96%). HER2 was occasionally expressed (*n* = 5/23, 22%) at a score of 2–3+. However, seven cases had diagnostic HER2 performed at the originating institutions. Six cases that were negative for HER2 on multiplex were positive for HER2 on uniplex diagnostic IHC (ranging from 1 + to 3+), and one additional case remained negative on uniplex. Therefore, up to 48% of cases (11/23) could be considered as expressing HER2. Diagnostic HER2 uniplex IHC was not performed on the remaining 11 multiplex negative cases. The possibility of false negative HER2 multiplex assay cannot be excluded on these cases.

All PAs with apocrine metaplasia had retained p40-positive myoepithelial cells in areas of apocrine epithelial metaplasia (*n* = 6/6, 100%). Most apocrine cells expressed androgen receptor (*n* = 5/6, 83%) but none expressed HER2 (*n* = 0/6, 0%).

All morphologically benign PAs demonstrated retained p40 in bilayered ducts (*n* = 41/41, 100%). Half (*n* = 21/41, 51%) showed androgen receptor expression in bland-appearing cells, including 18 in epithelial cells and 3 in myoepithelial cells. In 10 cases (including the 3 with myoepithelial cell expression), expression was very focal (< 5% of cells). None expressed HER2 (*n* = 0/41, 0%).

In SDC controls, cells within the pre-existing PA were scored separately from the invasive component. Seven of 8 cases had a pre-existing PA present on selected block for evaluation. In the pre-existing PA component, p40 was retained in 4/7 (57%), AR was positive in 6/7 (86%), and HER2 was positive (2–3+) in 4/7 (57%). In the invasive SDC component, p40 was retained in one case (*n* = 1/8, 13%), likely representing intraductal spread outside the confines of the PA. AR was positive in all (*n* = 8/8, 100%). HER2 was positive (2–3+) in over one-third (*n* = 3/8, 38%).

Overall, the rates of HER2 expression increased from benign PAs (with or without apocrine metaplasia, both 0%) to atypical PAs (22–48%) to SDC ex PA (38–57%). Similarly, the rates of AR expression increased from benign PAs (51%) to PAs with apocrine metaplasia (83%) to atypical PAs (96%) to SDC ex PA (86–100%) (Fig. 2).

## Literature Review (Table 3)

**Table 3:** Literature review

Article	*n*=	Outcomes	Average follow up (years)
LiVolsi 1977	6	No recurrence	N/A
Dolman 1986	1	No recurrence	1
Brandwein 1996	4	No recurrence	7.5
Lewis 2001	4	No recurrence	> 1.2
Felix 2002	1	*No recurrence	15.9
Di Palma 2005	11	No recurrence	2.2
Mensink 2005	1	No recurrence	6
Ethunandan 2006	3	No recurrence	2.2
Kunimura 2007	1	No recurrence	0.3
Logasundaram 2008	1	Recurrent benign PA, followed by myoepithelial carcinoma ex	12.9
Dandekar 2010	1	No recurrence	1
Mori 2010	3	No recurrence	4
Hashimoto 2011	20	No recurrence	4.3
Kim 2011	1	Not DOD	N/A
Weiler 2011	16	Not DOD	6
Matsushima 2012	1	Metastatic disease	0.3
Mariano 2013	1	No recurrence	9.6
Nakamura 2013	1	No recurrence	4
Griffith 2014	5	No recurrence	> 3
Mohan 2015	1	No recurrence	0.8
Nishijima 2015	14	Alive without metastases	N/A
Ismi 2015	1	No recurrence	1
Rito 2016	10	Metastatic disease	5.9
Sedassari 2017	6	No recurrence	N/A
Garakani 2020	1	No recurrence	0.8
Mori 2022	4	No recurrence	5
Song 2023	6	No recurrence	> 1.25
Fakhril-Din 2023	1	No recurrence	0.5
Quiroga 2023	1	No recurrence	1
Ren 2024	1	No recurrence	2
Alsugair 2024	2	No recurrence	1.6

*patient presented with metastases

The literature review resulted in 31 articles with a cumulative 130 cases that met inclusion criteria (Table 3). Additional detailed information is shown in supplementary Table 1. Reported diagnoses included PA with epithelial atypia, in situ CXPA, and intracapsular (either intraductal or extraductal) CXPA. No cases reported positive margins. The following metrics were found with the available reported data: The male to female ratio was 1.54:1. The average tumor size was approximately 3.2 cm. Sixty-six cases involved the parotid gland. Sixteen cases involved the submandibular gland. Four cases each involved the deep lobe parotid/parapharyngeal space, minor salivary glands, and lacrimal gland. One case arose in the preauricular area.

Average follow up was 4.2 (0.25–15.9) years. Six cases presented as a recurrent pleomorphic adenomas. All cases underwent surgical excision. Five cases received adjuvant radiation, and 2 cases received a neck dissection. Four cases reported disease progression: two had cervical lymph node metastasis [28, 29], one recurred as a benign pleomorphic adenoma and subsequently transformed into myoepithelial carcinoma [30], and one (which was already clinically recurrent) metastasized to the kidney with myoepithelial predominant morphology [31].

## Conclusion

Intracapsular/intratumoral or in situ CXPA (including PA with epithelial atypia/dysplasia) have been the subject of much discussion with some evidence supporting conservative treatment but little consensus on the degree of atypia, protein expression profile, or appropriate nomenclature that reflects biologic behavior. The subjective presence of intracapsular SDC or AEC reminiscent of SDC can be very worrisome, as SDC is the most common malignancy to arise in PA and has a very poor prognosis [2, 5]. This study overall showed no morphologic or immunohistochemical features that predicted poor outcomes in PAs with apocrine change or atypical/dysplastic/malignant epithelial cells when confined within the borders of the tumor. These findings call for a unified terminology that is reflective of this morphologic spectrum and good prognosis.

In concordance with current understanding, this study found that widely invasive SDC ex PA had high-grade morphology and poor prognosis, including the presence of necrosis (50%), > 1 mitosis/2 mm^2^ (75%), recurrence (63%, average 1.3 years after diagnosis), and disease-specific mortality (38%, average 2.3 years after diagnosis) even in the context of adjuvant chemoradiation. Conversely, the morphologically benign PAs, PAs with apocrine metaplasia, and PAs with any amount of SDC-like intratumoral epithelial atypia did not recur, metastasize, or cause death (average follow-up 10.5, 8.9, and 2.6 years, respectively). The strict inclusion criteria of negative margins likely contributed to the low recurrence rates in these groups.

Despite the good prognosis of PAs with AECs, there is no good morphologic consensus or guidelines for how much or what kind of cytologic changes warrant a diagnosis of “atypia,” “dysplasia,” or “carcinoma-in situ.” There is also no consensus on the prognostic significance of AR and/or HER2 expression in PAs of varying morphologies. It is well-known that salivary duct carcinoma often expresses AR and HER2 [25, 32, 33, 34, 35]. It is this expression pattern that has led many pathologists to a diagnosis of “salivary duct carcinoma in situ ex pleomorphic adenoma” in PAs that otherwise may have been called atypical. As expected, this study found the highest expression rates of AR and HER2 in the invasive SDC ex PA cohort, with AR and HER2 expression of 86% and 38% in the pre-existing PA and 100% and 57% in the invasive component, respectively. Still, malignant cases did not express both markers uniformly. Interestingly, one case showed HER2 positive cells within the pre-existing (partially burnt-out) PA but the invasive component was negative.

Cases with apocrine epithelial metaplasia without other malignant features (nuclear hyperchromasia, anisonucleosis, > 1 mitosis/2 mm^2^, or necrosis) as well as cases with AEC expressed AR at high rates (83% and 96%, respectively) and HER2 at low to moderate rates (0% and 22%, respectively). Completely morphologically bland PAs (negative control group) also expressed androgen receptor in 51% of cases, predominantly in the epithelial compartment, but did not express HER2. These findings are concordant with studies that have reported immunohistochemical expression of AR and HER2 in benign PAs (7–13% and 0–12%, respectively) [36, 37, 38]. Also, other studies have found preferential expression of AR and HER2 in AEC within PAs (53–93% and 0–81%, respectively) [18, 19, 20, 39, 40]. 

Additionally, most cases (91%) with AEC retained a p40-positive myoepithelial layer, compatible with the previously described intraductal intratumoral morphology. Only 2 (9%) lost p40 in atypical epithelial areas, compatible with extraductal intratumoral morphology [15]. All benign PAs and PAs with apocrine metaplasia retained p40. In contrast, approximately half of overt SDC ex PA showed p40 loss in the pre-existing PA, supporting the hypothesis that transformation progresses from escape of the myoepithelial cell layer prior to escape of the confines of the tumor. Interestingly, one SDC ex PA showed retention of p40 in the invasive component on the selected slide, perhaps reflecting spread within extratumoral, intraglandular ducts, supporting the concept of intraductal spread as part of the malignant progression of SDC ex PA [15, 41].

Apocrine metaplasia is a controversial phenomenon that may present a diagnostic dilemma, as there is some overlap between the morphology of AEC and apocrine metaplasia. It has even been suggested that apocrine changes may be a precursor to malignancy [20]. However, androgen receptor expression has been found in cells with apocrine morphology [42, 43] and is not necessarily predictive of poor behavior or cause for clinical concern. This concept is supported in the current literature, which does not suggest any correlation or worse prognosis. Lack of HER2 expression also supports that cells with apocrine morphology may simply represent metaplastic change. Various types of metaplasia are well-known in PAs, including mucinous, squamous, sebaceous, adipocytic, etc [6, 7, 8]. Therefore, the possibility that apocrine changes can exist in isolation as a benign, metaplastic process must be considered. Higher rates of AR expression as well as presence of HER2 expression and presence of mitoses/tumor necrosis in morphologically atypical PAs raise the possibility of a spectrum of transformation: morphologically bland with no evidence of apocrine change (AR negative) ->morphologically bland with molecular evidence of apocrine change (isolated AR expression) ->morphologic and immunophenotypic apocrine change (apocrine metaplasia and AR expression) ->increasing cytologic atypia (AR expression, necrosis, mitoses, possible HER2 expression) ->escape of the ductal system within the PA (intratumoral) ->escape of the confines of the PA (invasive).

In summary, this multi-institutional study identified 23 cases of histologically confirmed PAs with AEC, the largest cohort of its kind to date. PAs with AECs had rates of AR expression higher than benign and apocrine metaplastic counterparts. Additionally, they also had mitoses, tumor necrosis, and HER2 expression – all of which were lacking in benign and apocrine metaplastic counterparts. All cases had negative margins and no case recurred (compared to a 63% recurrence rate and 38% mortality rate of invasive SDC ex PA cases).

While a cause of diagnostic stress for practicing pathologists, pleomorphic adenomas with epithelial atypia/intracapsular carcinoma are rare. Literature review revealed 130 published cases that report follow up data [3, 15, 16, 19–21, 28–31, 39, 40, 44–61]. Four cases (3%) reported disease progression: metastasis to cervical lymph nodes (*n* = 2) or distant visceral sites (*n* = 1) or transformation to myoepithelial carcinoma ex pleomorphic adenoma (*n* = 1) [28, 29, 30, 31]. However, without evaluating the morphology of each of these cases, it is challenging to know if they would meet the criteria outlined by this study. Overall, the majority of reported cases (97%) showed no disease recurrence or progression.

The current study is also not without limitations. Cases with positive margins were excluded, as positive margins are well known to increase the recurrence potential of morphologically benign pleomorphic adenomas [26]. Consequently, the prognostic implications of a positive margin of a PA with AEC is outside the scope of this study, and further studies are indicated to address this question. Previous studies have found that minimally invasive SDC ex PA have potential for disease progression as both locoregional recurrence and metastasis [3, 15, 16]. This study also did not include minimally invasive SDC ex PA or other intracapsular lesions with non-epithelial atypia or non-SDC-like morphology. Also, in light of occasional discordance between multiplex HER2 performed for this study and uniplex HER2 performed in various diagnostic labs, the possibility of false negative HER2 in a subset of pleomorphic adenomas cannot be entirely excluded. However, as cases with positive expression did not have worse outcomes, the implications of false negative HER2 are likely low. Lastly, molecular testing was not performed but may shed light on possible similarities to invasive salivary duct carcinoma. However, next generation sequencing is in progress, and is anticipated to be presented in a subsequent manuscript.

Overall, this study presents the largest cohort of atypical PA / in situ CXPA to date, with morphologic, immunophenotypic, and clinical data. Data showed increasing average age from benign (~ 50 years) to atypical/in situ (60 years) to invasive carcinoma (~ 70 years), suggesting an average two-decade temporal pathway of transformation from benign to atypical to overtly malignant, with an increased frequency of transformation in males. Additionally, immunoexpression of androgen receptor does not appear to be specific to atypical/malignant PAs, as it was present in morphologically benign and apocrine metaplastic PAs. However, it was present at increased frequency in atypical and malignant PAs. In contrast, HER2 expression was restricted to a subset of atypical PAs and most SDC ex PA, perhaps serving as a marker of incipient transformation. However, neither morphology nor immunophenotype of atypical / in situ PAs predicted biologic behavior: all demonstrated indolent prognosis with negative margins.

In conclusion, based on the data in this study as well as a literature review, the presence of epithelial atypia within a pleomorphic adenoma (ranging from isolated AR expression to apocrine metaplasia to overtly dysplastic/malignant epithelial cells) does not portend aggressive behavior (recurrence or metastasis) if the atypia is confined within the borders of the adenoma and negative margins are achieved. While AR expression increased in conjunction with cytologic atypia and HER2 expression was reserved for overt epithelial dysplasia, these markers also did not impact prognosis. As malignant transformation is likely a morphologic spectrum and all intratumoral cases shared good prognosis, the exact cutoff that defines epithelial atypia compared to dysplasia or in situ carcinoma is ill-defined and likely arbitrary. Given the uniform indolent behavior, overdiagnosis of intratumoral AEC should be avoided and terminology should be employed that does not cause overtreatment. A diagnosis of “in situ / intracapsular / intratumoral salivary duct carcinoma ex pleomorphic adenoma” (whether intraductal / in situ or extraductal) may imply aggressive behavior and may lead to unjustified adjuvant chemo- or radiotherapy and unwarranted clinical concern. Therefore, use of this term is discouraged in light of the good prognosis. Alternatively, nomenclature such as pleomorphic adenoma with intratumoral epithelial “atypia” or “dysplasia” is recommended, followed by a comment regarding the morphologic features and likely indolent behavior.

The current literature in conjunction with results of the present study support invasion and margin status as the two most important factors in determining prognosis of a SDC ex PA. Consequently, PAs with AECs (including apocrine metaplasia and any amount of epithelial dysplasia) should be treated conservatively with complete excision with negative surgical margins and clinical follow up without neck dissection and/or adjuvant chemoradiation.

## Data Availability

No datasets were generated or analysed during the current study.

## Declarations

Matt Levin and David Ferber are employees of Leica Biosystems.
