# Correction: Pleomorphic Adenoma with Epithelial Atypia, Apocrine Metaplasia, and/or In situ/Intracapsular Salivary Duct Carcinoma Are Indolent Lesions with Good Prognosis: A Proposal for Unified Nomenclature and Clinical Observation

**DOI:** 10.1007/s12105-025-01866-z

**Published:** 2026-05-29

**Authors:** Grayson G. Cole, Matt Levin, David Ferber, Spencer C. Roark, Peter M. Sadow, Daniel Lubin, Julie Guilmette, Jason R. Pettus, Adam S. Fisch, Dipti P. Sajed, Fouad R. Zakka, Mark W. Lingen, Nicole A. Cipriani

**Affiliations:** 1 Department of Pathology, University Hospitals, Cleveland, OH USA; 2 Cell IDx, Inc. (A Division of Leica Biosystems), San Diego, CA USA; 3 Medical & Scientific Affairs, Leica Biosystems, Deer Park, IL USA; 4 Vanderbilt University Medical Center, Nashville, TN USA; 5 Department of Pathology, Massachusetts General Hospital, Harvard Medical School, Boston, MA USA; 6 Emory Healthcare, Atlanta, GA USA; 7 Hôpital Charles-Le Moyne, Québec, QC USA; 8 Dartmouth-Hitchcock Medical Center, Lebanon, NH USA; 9 Department of Pathology, University of California, Los Angeles, Los Angeles, CA USA; 10 Wellstar Health System, Atlanta, GA USA; 11 Department of Pathology, The University of Chicago, Chicago, IL USA

**Correction to: Head and Neck Pathology (2025) 19:109** 10.1007/s12105-025-01841-8.

A part of the first author’s name “Cole” was repeated. It is now removed. The original article has been corrected.

